# A Study on the Mechanism and Strategy of Cross-Regional Emergency Cooperation for Natural Disasters in China—Based on the Perspective of Evolutionary Game Theory

**DOI:** 10.3390/ijerph182111624

**Published:** 2021-11-05

**Authors:** Jida Liu, Yanan Guo, Shi An, Chenxi Lian

**Affiliations:** School of Management, Harbin Institute of Technology, Harbin 150001, China; kittadada@yeah.net (J.L.); 13804551661@163.com (Y.G.); anshi@hit.edu.cn (S.A.)

**Keywords:** natural disasters, emergency management, evolutionary game, cross-regional, emergency cooperation

## Abstract

Natural disasters have obvious cross-regional and compound characteristics. Cross-regional emergency cooperation for natural disasters deepens the diversification of coordination relations and the complexity of interaction modes among emergency response organizations, including horizontal and vertical organizational interactions. In order to clarify the cooperation mechanism of emergency organizations during cross-regional emergency cooperation for natural disasters and to explore the key factors that affect the cooperative relationships of emergency organizations, in this study, a game model is constructed based on evolutionary game theory, which is composed of local, neighboring, and central governments. Then, the stability of the emergency game strategy is analyzed. On this basis, a numerical simulation is used to simulate the dynamic evolution trajectory of the game system. The results show that there is an embedded mutual promotion mechanism that evolves towards a positive emergency strategy combination among the game subjects. The selection strategies of the game subjects show the characteristics of consistency and the following: enhanced cooperation efficiency between local and neighboring governments, emergency capital stock, and shared resources, therefore, guiding social emergency forces to actively participate in emergency operations. Strengthening the emergency dispatching strength of the central government and the effectiveness of central–local emergency dispatching, can support the performance of cross-regional emergency cooperation for natural disasters. Furthermore, the efficiency of cooperation between local and neighboring governments will be enhanced.

## 1. Introduction

A natural disaster is an important factor that restricts a country’s economic and social development and affects the safety of people’s lives and property [1]. China is one of the countries most affected by natural disasters in the world [2,3]. Meteorological, earthquake, geological, flood, and marine disasters, as well as forest and grassland fires are the main types of natural disasters in China [4,5]. The main characteristics of natural disasters in China include the multiple types of disasters, wide distribution areas, high frequency of occurrence, heavy disaster losses, and strong disaster risks [6]. At the same time, with a rapid increase in accidents and disasters caused by the global COVID-19 pandemic and an accelerated urbanization process, intertwining and superimposing natural disasters and other types of risks have become prominent, and the number of groups and chain emergencies has increased [7,8]. The government of China will face more complex and severe situations and challenges in dealing with natural disasters.

At this stage, due to the characteristics of development uncertainty, external complexity, evolutionary dynamics, and unpredictable trends of natural disasters, a single emergency organization cannot effectively respond to and meet the various needs necessary to handle natural disasters [9]. There is an urgent need for further coordination and cooperation among emergency organizations at different levels, scales, and types. From a practical point of view, strengthening the coordination mechanism between governments and other emergency organizations is a basic requirement of the world’s response to natural disasters [10]. Effective coordination and communication between different types of emergency organizations are necessary conditions for improving the effectiveness of natural disaster emergency management [11]. Effective cooperation between emergency organizations avoids wasting resources and reduces costs in the process of a natural disaster emergency response, and further improves the efficiency of emergency operations based on a solidified trust relationship between emergency organizations [12]. In order to effectively respond to various natural disasters, to improve the emergency organization and command system, and to fully embrace the functions of various departments in the prevention and control of natural disasters, the government of China has established cross-sectorial coordination models, such as deliberative and coordinating agencies and inter-ministerial joint meetings at both the national and local levels [13].

More and more, emergency cases have shown that natural disasters have significant cross-regional characteristics [14]. After the occurrence of a natural disaster, the development and spread of the disaster and a government’s off-site case handling and emergency rescue when responding are the main connotations of cross-regional characteristics of natural disasters. Cross-regional emergency cooperation is an important governance measure [15]. Cross-regional emergency cooperation for natural disasters deepens the diversification of coordination relations among emergency management organizations and the complexity of interaction modes [16,17].

In China, government departments and non-government organizations (NGO) with different levels and functions usually constitute the overall overview of emergency response organizations. Government departments can be divided into central and territorial governments according to administrative levels, and can also be divided into command, rescue, and guarantee organizations according to functions. Correspondingly, social organizations can be divided into private organizations, public welfare associations, and volunteer rescue teams. In addition, civil NGOs, government-organized NGOs, and international NGOs can be classified according to the form of ownership. In terms of cooperation mode, cross-regional emergency cooperation includes cooperation among governments in different administrative regions, relevant functional departments, and governments with social organizations in the horizontal dimension, as well as the cooperation and coordination of governments and departments at different levels in the vertical dimension [18]. In contrast, clarifying the cross-regional emergency cooperation mechanism of natural disasters and identifying the key factors affecting the relationships among emergency organizations are of great value in order to improve the level of cross-regional emergency cooperation. The different characteristics of the different levels of government and functional departments, in addition to different decision-making approaches in cross-regional emergency cooperation among governments, are also ignored to some extent.

Therefore, through the practice of natural disaster emergency management in China, in this study, we construct a game model of cross-regional emergency cooperation for natural disasters. The game model is composed of three agents: the local government, the neighboring government, and the central government. The stabilities of the strategy’s game subjects are analyzed by identifying the different attributes of cross-regional emergency cooperation for natural disasters. On this basis, the dynamic evolution trajectory of cross-regional emergency cooperation for a natural disaster game system is simulated through a numerical simulation. The strategy selection law and development paths of the local, neighboring, and central governments in cross-regional emergency cooperation are clarified and interpreted. Further, countermeasures, suggestions, and solutions are put forward in order to break through the bottleneck of cross-regional emergency cooperation and to optimize the cross-regional emergency cooperation mechanism, and therefore to reduce the losses and negative impacts of natural disasters on social security and economic development.

The framework of this study is as follows: In Section 2, we search previous study results and review the status of the research on the two dimensions of emergency cooperation and cross-regional emergency management. Furthermore, the necessity of this study is introduced. In Section 3 we describe the research problem. The model hypothesis and parameter settings are presented. The payment matrix and the replication dynamic equation of the three-agent game subjects under different strategy combinations are solved. In Section 4, we explore the strategic stability of the local, neighboring, and central governments, which is combined with the stability theorem of the differential equation and the phase diagram of the game subjects’ strategies. In Section 5, a numerical simulation analysis is applied in order to describe the dynamic evolution behavior of the local, neighboring, and central governments in cross-regional emergency cooperation for natural disasters. Furthermore, the influence mechanism of important parameter changes on the strategy evolution of the game subjects is examined. In Section 6, we discuss the study results and provide possible development directions for the next steps of the research. In Section 7, we summarize our conclusions and put forward policy considerations that are in line with the reality of cross-regional emergency cooperation for natural disasters.

In general, the potential contributions of this study include: (1) The introduction of evolutionary game theory into the problem of cross-regional emergency cooperation for natural disasters, which provides an observable window for exploring horizontal and vertical intergovernmental emergency cooperation models. (2) A description of the cooperative relationships among agents, social capital theory, dissipative structure theory, and signaling theory are introduced. Variable parameters are set, such as the coverage effect coefficient of emergency capital, the efficiency coefficient of emergency rescue cooperation, the response coefficient of social emergency forces, the effective coefficient of national–local government emergency dispatching, the emergency dispatching degree of the central government, and the matching degree of cross-regional emergency rescue. The above variable parameters form multiple perspectives in order to describe the cooperation modes among game subjects. (3) We combine the results of the numerical simulations, and discuss a practical path for cross-regional emergency cooperation systems for natural disasters to evolve to a combination of active and positive strategies. Countermeasures and suggestions for improving the level of cross-regional emergency management of natural disasters are provided. Our results also have theoretical and practical values for governments for improving the cross-regional emergency cooperation mechanism of natural disasters.

## 2. Literature Review

A common pursuit of governments around the world is to reduce the impact of and to improve the relief system for natural disasters. To understand the basic connotation and theoretical origin of cross-regional emergency cooperation for natural disasters, it is necessary to discuss this in detail from two dimensions: emergency cooperation and cross-regional emergency management.

### 2.1. Emergency Cooperation

In recent years, research on emergency cooperation has been an increasing concern of the academic community and has achieved rapid development. The existing literature in China has fully affirmed the key role of cooperation in emergency preparedness and emergency response in improving the performance of emergency management and reducing the loss caused by emergencies. 

For the emergency preparedness cooperation phase, governments at all levels, functional departments, and social organizations form certain cooperation norms, trust basis, and relevance through the preparation of contingency plans, emergency preparedness, emergency drills, and past emergency cooperation. Previous studies have applied social capital theory to describe the attributes of this relationship [19]. Social capital is a collection of actual or potential resources that exist in the social structure, which are different from physical capital and human capital, and can provide convenience for actors within the structure [20,21]. According to social capital theory, the maintenance of organizational cooperation is not limited to the reserves of resources owned by the organization, but depends more on the accessibility of resources that flow across the organization. Therefore, in this study, we introduce social capital theory to express the series of relations formed in the emergency preparedness cooperation phase as emergency capital stock. Cooperation norm, trust basis, and association relation will correspond to institutional norm capital stock, trust relation capital stock, and information matching capital stock, respectively.

For the emergency response cooperation phase, in this study, we focus on exploring the characteristics of emergency cooperation in different emergencies, the role of various emergency organizations in emergency cooperation, and the unique characteristics of different emergency cooperation links. Regarding disaster types of emergency cooperation, existing studies have explored organizational cooperation modes in the emergency response processes of earthquakes, hurricanes, floods, production safety accidents, public health emergencies, terrorist attacks, and social security incidents. Additionally, the typical characteristics of emergency organization cooperation in different emergencies have been further analyzed. The differences between organizational cooperation and institutional provisions in emergency response and emergency preparedness have also been compared. From the perspective of the participants in emergency cooperation, the coordination and interaction of emergency management in China occurs between government departments [22], as well as between government and social organizations and between government and military [23]. To be specific, the intergovernmental emergency cooperation mode includes cooperation among local central governments, cooperation among different local governments, cooperation among different government departments, and cooperation among trans-central governments [24]. In China, social organizations involved in emergency cooperation mainly include non-profit groups, volunteer associations, volunteer emergency rescue teams, and other non-governmental organizations (NGOs) [25]. From the implementation of the emergency cooperation, related scholars have launched detailed discussions concerning emergency rescue cooperation, resource support cooperation, security regulatory cooperation, warning issued cooperation, and post-disaster recover [26]. Meanwhile, through the system design of the Chinese government, feedback from emergency practical experience, and multiple other perspectives, the construction ideas and implementation suggestions for strengthening the organizational cooperation mechanisms in different emergency departments and social organizations were summarized.

In addition, in order to more clearly analyze the types of emergency organizations and to discuss the interactions and collaboration relationships of emergency organizations in emergency cooperation, studies have introduced the network research method in order to analyze the scale of emergency organization cooperation, the overall form, and the dynamic mechanism of the emergency organization network from practical emergency cases [11,27]. At the same time, because abruptness, dynamics, and uncertainty are important characteristics of organizations in response to emergencies, other studies have been conducted on the dynamic emergency cooperation network from the perspectives of network evolution in different periods or network comparisons before and after emergencies. This solves the dynamic evolution law of emergency cooperation networks, and also explores the similarities and differences between the target state and practice mode of government emergency cooperation, which provides a traceable analysis path for the practical feedback learning mechanism of emergency management [28].

Research on emergency cooperation has gradually formed a great wealth of study systems and theoretical contexts. However, at the same time, with the further increase in the types of unconventional emergencies and risk incentives, the phenomenon of mass or chain occurrences of multiple types of emergencies is prominent. Compound emergency management has become a major challenge and an emerging topic addressed by theoretical research institutions and government departments in recent times. Currently, in breaking the boundary of previous emergency cooperation modes, emergency cooperation has expanded in subject type, cooperation scale, and interaction mode. The relationship between emergency organizations is more complex. From the perspective of thermodynamics, the emergency system formed based on the cooperation of emergency organizations is a process of increasing entropy [29]. Due to the urgency, suddenness, and uncertainty of emergencies, emergency organizations will gradually move to the state of “disorder”. Therefore, it is necessary to introduce negative entropy flow to realize the order and organization of emergency systems. A description of the process of emergency systems by dissipative structure theory has proven to be feasible [30].

### 2.2. Cross-Regional Emergency Management

Cross-regional governance is an innovative governance model that breaks the traditional management and control governance model that is confined to the boundaries of administrative divisions [31,32]. Therefore, governments, enterprises, the public, and NGOs have achieved comprehensive interactive and cooperative governance. As an innovative governance model used to solve governance dilemmas of complex public affairs and to meet the needs of regional coordinated development, governments around the world have actively explored and formed a series of distinctive new governance practices in environmental governance [33], economic development [34], traffic management [35], energy management [36], waste management [37], public health [38], and other fields. It can be concluded that cross-regional governance has become an effective governance model used to solve cross-regional public affairs and to realize regional sustainable development.

As one of the important dimensions of government cross-regional governance, the implementation of cross-regional emergency management contributes to the effective operation of emergency management as a complex adaptive system. At present, paying attention to the cross-regional characteristics of emergencies has become an important guide in the field of emergency management. The Emergency Management Assistance Compact in the United States is often used as a typical example of cross-regional cooperation in emergency management. The Chinese government’s practice of carrying out cross-regional emergency cooperation for natural disasters mainly includes two modes. Firstly, local governments have actively explored regional coordinated development. Governments at the same level have deployed intergovernmental cooperation in natural disaster emergency responses through signing cooperation agreements and implementing joint emergency drills. Secondly, there has been passive adaptation of local governments to the emergency needs of natural disasters. The government conducts emergency support operations through emergency force transmission and emergency material deployment during periods of emergency response, recovery, and reconstruction [39,40]. In the Pearl River Delta, the Beijing–Tianjin–Hebei Region, and the Yangtze River Delta, China has initiated the practice and exploration of cross-regional emergency management cooperation. Most scholars take the actual cases of emergencies as the starting point to preliminarily discuss the cross-regional attributes of different types of emergencies. The core characteristics of cross-regional emergency management, which have been widely recognized, are fluidity, extensibility, causality, and systematicness. Specifically, mobility of air pollution and waste discharge, transmission of public health emergencies, spread of natural disasters, occurrence of secondary disasters, and inducement of safety production accidents are all externalized manifestations of the cross-regional characteristics of emergencies. At present, the relevant topics of cross-regional emergency management research usually focus on distribution and coverage of emergency forces [41], dispatch and allocation of emergency resources [42], site selection of emergency rescue stations [43], transmission of emergency command [44], and dispatch of the cross-regional traffic networks.

An in-depth analysis of the decision-making mechanisms of a government and relevant departments in response to emergencies is consistently appealing to improve governments’ emergency management capacities. Predictably, the intergovernmental decision-making mechanism involved in cross-regional emergency cooperation for natural disasters is interactive and dynamic. The differential carrier of measuring the decision-making mechanism is information transmission between governments. In order to clearly describe the information transmission mechanism of intergovernmental emergency decision making, in this study, we introduce signaling theory to express the information content of emergency command decision making and emergency operation feedback. Signaling theory is usually used to explain information asymmetry between organizations in different situations [45], which has strong explanatory power for the information asymmetry of emergency decision making caused by hierarchical relationship and function distribution [46].

Exploring different levels and cooperative relations among the regional governments is very valuable for understanding cross-regional emergency cooperation for natural disasters. In this study, we draw on evolutionary game theory in order to describe the interactions between emergency organizations in the cross-regional emergency cooperation for natural disasters. In recent years, evolutionary game theory has also been applied and developed in emergency management problems [47,48], such as the prevention and control of public health emergencies [49,50], government safety supervision [51], guiding emergency mass evacuation [52], government emergency mobilization [53], and emergency handling strategies of engineering construction [54]. The dynamic decision-making mechanism of different emergency organizations has been examined based on the strategy types and evolution process of participants in emergency management. The academic community has also tried to further explain the influencing factors of the change in emergency organization strategy from the perspectives of the vertical administrative constraint mechanism, reward and punishment mechanism, public participation, and preferential emergency policy. In terms of the selection of game subjects in the cross-regional emergency cooperation for natural disasters, in this study, we focus on the horizontal cooperative relationships among different regional governments, and also on the vertical interaction between the central and local governments.

## 3. Research Problem Description and Basic Assumptions

### 3.1. Problem Description

After a natural disaster occurs, as the main subject responsible for the prevention and treatment of natural disasters, governments need to respond to natural disaster emergency needs in a coordinated manner. At the same time, government should coordinate emergency rescue forces to participate in emergency support, guide the coordination among the emergency subjects, and realize the orderly allocation of emergency resources. At present, in order to effectively deal with the cross-regional characteristics of natural disasters, cross-regional emergency cooperation modes have been formed among governments at different levels and regions.

In the cross-regional emergency cooperation for natural disasters, there are the central government’s emergency commands to the local government and the local government’s instruction feedback to the central government, as well as the cooperation and interaction between the local government and its neighboring government. Therefore, the local government in charge during a natural disaster, which has the responsibility for the emergency response and emergency rescue, will perform the main responsibilities of on-site command and emergency treatment. The local government should assume the responsibility of guiding the coordination and orderly participation of various emergency organizations. In the face of a natural disaster, in order to reduce possible losses to the region, and in order to carry out the cross-regional emergency cooperation agreement and to receive the dispatch instructions from the central government, the neighboring government needs to cooperate with the local government to implement emergency actions. The central government undertakes the work of the national headquarters for responding to particularly major natural disasters. During a natural disaster, the central government should coordinate the unified dispatch of emergency forces and emergency resources. At the same time, it is necessary to guide the local government in relation to the implementation of emergency rescue and disaster relief.

In addition, emergency cooperation strategies of the game subjects are influenced by horizontal coordination among emergency organizations, matching of rescue forces, and adaptation of cooperation willingness, and they are also affected by vertical administrative orders, resource scheduling, and command information transmission. The strategic choices of local, neighboring, and central governments in the cross-regional emergency cooperation for natural disasters will determine the effectiveness of natural disaster emergency management. Therefore, the focus of this study is to clarify the influencing factors and evolutionary mechanism of the strategic choice behavior of local, neighboring, and central governments.

### 3.2. Assumptions and Parameter Settings

**Figure 1:**
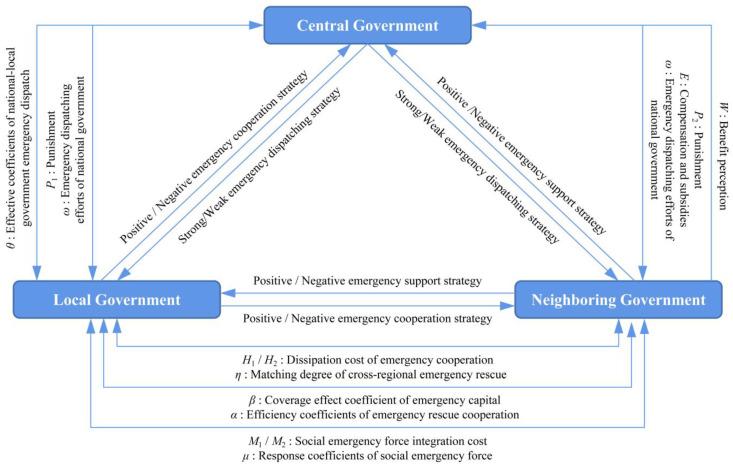
The game relationship between local, neighboring, and central governments.

In this study, we built a three-agent evolutionary game model of local, neighboring, and central governments in the cross-regional emergency cooperation for natural disasters, as shown in Figure 1. In order to clarify the dynamic selection mechanism of emergency strategies for the local, neighboring, and central governments in cross-regional emergency cooperation, the following assumptions should be made. The definition and basic description of the parameters involved in the model assumptions are shown in Table 1.

**Assumption** **1.** *In the game model of cross-regional emergency cooperation for natural disasters, the government decision-makers of the local, neighboring, and central governments all have the characteristics of bounded rationality. In the game model of cross-regional cooperation in an emergency, there is information asymmetry among the local, neighboring, and central governments. The game strategy choice among the three agents is randomly paired and repeated.*

**Assumption** **2.** *In the game process of cross-regional emergency cooperation for natural disasters, the local government can choose a positive emergency cooperation strategy in order to enhance the efficiency of the emergency and to reduce the loss caused by natural disasters. The local government can choose a negative emergency cooperation strategy in order to reduce the emergency cost. The probability of a positive and a negative emergency cooperation strategy chosen by the local government is x and 1 − x (0 ≤ x ≤1), respectively. The neighboring government can choose a positive emergency support strategy to improve the level of cooperation with the local government and strengthen the investment in emergency response, and therefore reduce the possible damage of natural disasters to neighboring areas. At the same time, the neighboring government may be limited by the problems of funding sources, emergency force, and the mentality of not suffering from disaster losses, and then chooses a negative emergency support strategy to reduce the emergency cost. The probability of a positive and negative emergency support strategy selected by the neighboring government is y and 1 − y (0 ≤ y ≤ 1), respectively. The central government can adopt a strong emergency dispatching strategy to improve the command and supervision level of the local government and the neighboring government, to improve the emergency response efficiency and further reduce the losses caused by natural disasters. However, the central government may be limited by the total amount of emergency resources or unwilling to pay more emergency costs, and then chooses a weak emergency dispatching strategy. The probability corresponding to a strong and weak emergency dispatching strategy is z and 1 − z (0 ≤ z ≤ 1), respectively.*

**Assumption** **3.** *Natural disasters cause heavy losses to economic and social development as well as people’s lives and property. In the game model of cross-regional emergency cooperation for natural disasters, the total perception of the local, neighboring, and central governments on the loss of human casualties, industry damage losses, and social development losses caused by natural disasters and derivative disasters are L*_1_*, L*_2_*, and L*_3_*, respectively. At the same time, in order to reduce the losses caused by natural disasters, the local, neighboring, and central governments all need to pay corresponding emergency response costs. Emergency response costs mainly include organization and management costs, coordination and communication costs, emergency force costs, and emergency goods and materials. Therefore, the emergency response costs of the local, neighboring, and central governments are C*_1_*, C*_2_*, and C*_3_*, respectively. In addition, when carrying out emergency response operations on natural disasters, the government will gain public credibility and reputation benefits from society. The public benefits of the local, neighboring, and central governments are R*_1_*, R*_2_*, and R*_3_*, respectively.*

**Assumption** **4.** *In order to consolidate the emergency management responsibility of the local government and the neighboring government, the central government will evaluate the emergency response operations of the local and neighboring governments. Then, the central government will punish the local and neighboring governments that choose a negative emergency strategy. The corresponding punishment is P*_1_* and P*_2_*, respectively. When the neighboring government chooses a positive emergency support strategy, the central government will obtain the benefit perception W formed by the reduction of natural disaster losses. At the same time, the central government will provide certain financial compensation and supply subsidies for the cost E of the emergency response, which was paid by the neighboring governments by adopting positive emergency support strategies.*

In order to measure the central government’s intensity of participation in an emergency response and the supervision of the local government participation in an emergency response, we propose the emergency dispatching degree of the central government *ω*. When the intensity of the central government’s emergency dispatching is greater, the central government will increase the cost of emergency response, the intensity of administrative accountability punishment, and the intensity of emergency compensation and reward. Meanwhile, when the central government chooses a strong emergency dispatching strategy, *ω* is going to be 1. Therefore, the cost of the emergency response paid by the central government under a weak emergency dispatching strategy is *ωC*_3_. The accountability punishments for the local and neighboring governments are *ωP*_1_ and *ωP*_2_, respectively, and the compensation for the neighboring government is *ωE*.

**Assumption** **5.** *It is thought that entropy increases in a system of cross-regional emergency cooperation for natural disasters [29]. In other words, under a positive emergency strategy, the local and neighboring governments will encounter problems, such as temporarily adding new emergency tasks, “disorderly” collecting emergency forces, and poorly transmitting the emergency information [55]. According to dissipative structure theory, the local and neighboring governments need to pay additional emergency response costs to dissipate this “entropy increase”. Therefore, the concept of dissipative cost of emergency cooperation was proposed. The dissipative costs of emergency cooperation between local and neighboring governments is H*_1_* and H*_2_*, respectively.*

On this basis, we can describe the tacit understanding degree between the local and neighboring governments in emergency cooperation. In this study, the matching degree of cross-regional emergency rescue *η* is introduced. Under a positive emergency strategy, the higher *η* is, the better the matching degree and adaptability of task assignment, organization coordination, and information interaction are between the local and neighboring governments. The dissipative cost of emergency cooperation can be avoided more effectively.

**Assumption** **6.** When a natural disaster occurs, all subjects in the social system have a certain willingness to participate in emergency response. As an important supplement to a government’s emergency response system, the social emergency forces are key forces for assisting and cooperating with government departments in response to natural disasters and conducting rescue operations. At this time, a local government and a neighboring government that choose a positive emergency strategy should integrate and orderly guide social emergency forces to participate in emergency response operations. The local and neighboring governments should pay the organization and management costs, M*_1_* and M*_2_*, respectively. However, the enthusiasm and response level of social emergency forces in different regions and at different levels are obviously different. Therefore, in order to measure the level of social emergency forces supporting a government’s emergency response operations, we propose the response coefficient of social emergency forces μ. When the response coefficients of the social emergency force are larger, the cost of integrating social emergency forces of the local government and the neighboring government will be smaller, which is (1 − μ)*M*_1_* and (1 − μ)*M*_2_*, respectively.

**Assumption** **7.** *Social capital theory proposes that individuals, groups, and organizations will obtain tangible and intangible social capital resources through social interaction [21]. In emergency cooperation, emergency organizations can form emergency capital stock based on institutional norms, trust relationships, and information matching during cross-regional emergency cooperation, cross-regional emergency agreement and plan preparation, and natural disaster forecast consultation [56]. The same is true for the local and neighboring governments in the emergency cooperation for natural disasters. Emergency capital can measure the size of fixed and formed capital resources among emergency organizations. Therefore, the emergency capital stock will affect the emergency response costs paid by the local and neighboring governments. The high accumulation of emergency capital stock means that the local and neighboring governments will pay less during the actual emergency response.*

Therefore, in this study, we introduce the coverage effect coefficient of emergency capital *β* between the local and neighboring governments to measure the emergency capital stock that can be invoked by the government under a positive emergency strategy. The high-level *β* means that the local and neighboring governments can offset more emergency response costs with the emergency capital stock in the actual emergency response. When both the local and neighboring governments choose a positive emergency strategy, the emergency response costs to be paid are (1 − *β*)*C_1_ and (1 − *β*)*C_2_, respectively.

**Assumption** **8.** *In this study, we introduce the efficiency coefficient of emergency rescue cooperation α as a variable to evaluate the implementation effect of emergency operations by local and neighboring governments with limited emergency resources. When both the local and neighboring governments choose a positive emergency strategy, the higher α indicates that they have a better level of resource sharing, equipment sharing, and information transmission. The local and neighboring governments have higher emergency response efficiency, which can reduce natural disaster losses and the dissipative cost of emergency cooperation.*

**Assumption** **9.** *The signaling theory is usually introduced to explain information asymmetry between organizations in different situations [45]. Information asymmetry also exists between the central government and local governments in the emergency cooperation of natural disasters. At the same time, the central government’s emergency command and the local government’s emergency response feedback are the important factors affecting the implementation of emergency relief action. Therefore, in order to describe the effectiveness of the central government’s dispatch to the local government’s emergency response operations, we propose the effective coefficient of national-local government emergency dispatching θ. When the central government chooses a strong emergency dispatching strategy and the local government chooses a positive strategy, a high level of θ means that the central government’s emergency dispatching command is more effective, the local government’s response feedback of emergency operations is clearer, and the government’s various losses due to natural disasters are lower.*

### 3.3. Payment Matrix

On the basis of the above assumptions and the game situation of the local, neighboring, and central governments under the cross-regional emergency cooperation mechanism for natural disasters given by the research, the game payment matrix of the three agents under different strategy combinations can be obtained, as shown in Table 2 and Table 3.

### 3.4. Replication Dynamic Equations of Each Game Subject

#### 3.4.1. The Local Government

The expected benefit of the positive emergency cooperation strategy selected by the local government *U*_11_ is as follows:

(1) $$\begin{array}{l} U_{11}=yz\left[-\left(1-\beta \right)C_{1}-\left(1-\eta \right)\left(1-\alpha \right)H_{1}-\left(1-\theta \right)\left(1-\alpha \right)L_{1}+R_{1}-\left(1-\mu \right)M_{1}\right]+y\left(1-z\right)\left[-\left(1-\beta \right)C_{1}-\left(1-\eta \right)\left(1-\alpha \right)H_{1}-\left(1-\alpha \right)L_{1}+R_{1}-\left(1-\mu \right)M_{1}\right]\\ +z\left(1-y\right)\left[-C_{1}-\left(1-\eta \right)H_{1}-\left(1-\theta \right)L_{1}+R_{1}-\left(1-\mu \right)M_{1}\right]+\left(1-y\right)\left(1-z\right)\left[-C_{1}-\left(1-\eta \right)H_{1}-L_{1}+R_{1}-\left(1-\mu \right)M_{1}\right] \end{array}$$

Then, the expected benefit of the negative emergency cooperation strategy selected by the local government *U*_12_ is as follows:

(2) $$U_{12}=yz\left(-C_{1}-L_{1}-P_{1}\right)+y\left(1-z\right)\left(-C_{1}-L_{1}-\varpi P_{1}\right)+z\left(1-y\right)\left(-C_{1}-L_{1}-P_{1}\right)+\left(1-y\right)\left(1-z\right)\left(-C_{1}-L_{1}-\varpi P_{1}\right)$$

The average expected benefit of the local government’s emergency cooperation strategy is:

(3) $$\bar{U_{1}}=xU_{11}+\left(1-x\right)U_{12}$$

Therefore, the replication dynamic equation of the local government’s emergency cooperation strategy is:

(4) $$F\left(x\right)=\frac{dx}{dt}=x\left(U_{11}-\bar{U_{1}}\right)=x\left(1-x\right)\left[R_{1}-\left(1-\mu \right)M_{1}+\varpi P_{1}+z\left(1-\varpi \right)P_{1}+y\beta C_{1}-\left(1-y\alpha \right)\left(1-\eta \right)H_{1}+\left(y\alpha +z\theta -yz\alpha \theta \right)L_{1}\right]$$

Furthermore, the derivation of the replication dynamic equation of the local government’s emergency cooperation strategy is obtained:

(5) $$F^{\prime }\left(x\right)=\left(1-2x\right)\left[R_{1}-\left(1-\mu \right)M_{1}+\varpi P_{1}+z\left(1-\varpi \right)P_{1}+y\beta C_{1}-\left(1-y\alpha \right)\left(1-\eta \right)H_{1}+\left(y\alpha +z\theta -yz\alpha \theta \right)L_{1}\right]$$

#### 3.4.2. The Neighboring Government

Likewise, the expected benefit of the positive emergency support strategy selected by the neighboring government *U*_21_ is as follows:

(6) $$\begin{array}{l} U_{21}=xz\left[-\left(1-\beta \right)C_{2}-\left(1-\eta \right)\left(1-\alpha \right)H_{2}-\left(1-\alpha \right)L_{2}+R_{2}-\left(1-\mu \right)M_{2}+E\right]+x\left(1-z\right)\left[-\left(1-\beta \right)C_{2}-\left(1-\eta \right)\left(1-\alpha \right)H_{2}-\left(1-\alpha \right)L_{2}+R_{2}-\left(1-\mu \right)M_{2}+\varpi E\right]\\ +z\left(1-x\right)\left[-C_{2}-\left(1-\eta \right)H_{2}-L_{2}+R_{2}-\left(1-\mu \right)M_{2}+E\right]+\left(1-x\right)\left(1-z\right)\left[-C_{2}-\left(1-\eta \right)H_{2}-L_{2}+R_{2}-\left(1-\mu \right)M_{2}+\varpi E\right] \end{array}$$

Then, the expected benefit of the negative emergency support strategy selected by the neighboring government *U*_22_ is as follows:

(7) $$U_{22}=xz\left(-C_{2}-L_{2}-P_{2}\right)+x\left(1-z\right)\left(-C_{2}-L_{2}-\varpi P_{2}\right)+z\left(1-x\right)\left(-C_{2}-L_{2}-P_{2}\right)+\left(1-x\right)\left(1-z\right)\left(-C_{2}-L_{2}-\varpi P_{2}\right)$$

The average expected benefit of the neighboring government’s emergency support strategy is:

(8) $$\bar{U_{2}}=yU_{21}+\left(1-y\right)U_{22}$$

Therefore, the replication dynamic equation of the neighboring government’s emergency support strategy can be obtained as:

(9) $$G\left(y\right)=\frac{dy}{dt}=y\left(U_{21}-\bar{U_{2}}\right)=y\left(1-y\right)\left[R_{2}-\left(1-\mu \right)M_{2}+\varpi \left(E+P_{2}\right)+z\left(1-\varpi \right)\left(E+P_{2}\right)+x\beta C_{2}-\left(1-x\alpha \right)\left(1-\eta \right)H_{2}+x\alpha L_{2}\right]$$

In the next step, the derivation of the replication dynamic equation of the neighboring government’s emergency support strategy is obtained as:

(10) $$G^{\prime }\left(y\right)=\left(1-2y\right)\left[R_{2}-\left(1-\mu \right)M_{2}+\varpi \left(E+P_{2}\right)+z\left(1-\varpi \right)\left(E+P_{2}\right)+x\beta C_{2}-\left(1-x\alpha \right)\left(1-\eta \right)H_{2}+x\alpha L_{2}\right]$$

#### 3.4.3. The Central Government

For the central government, the expected benefit of choosing a strong emergency dispatching strategy is:

(11) $$U_{31}=xy\left[-C_{3}-\left(1-\theta \right)L_{3}+R_{3}+\theta W\right]+x\left(1-y\right)\left[-C_{3}-\left(1-\theta \right)L_{3}+R_{3}\right]+y\left(1-x\right)\left(-C_{3}-L_{3}+R_{3}+\theta W\right)+\left(1-x\right)\left(1-y\right)\left(-C_{3}-L_{3}+R_{3}\right)$$

Then, the expected benefit of a weak emergency dispatching strategy selected by the central government is:

(12) $$U_{32}=xy\left(-\varpi C_{3}-L_{3}\right)+x\left(1-y\right)\left(-\varpi C_{3}-L_{3}\right)+y\left(1-x\right)\left(-\varpi C_{3}-L_{3}\right)+\left(1-x\right)\left(1-y\right)\left(-\varpi C_{3}-L_{3}\right)$$

The average expected benefit of the central government’s emergency dispatching strategy is:

(13) $$\bar{U_{3}}=zU_{31}+\left(1-z\right)U_{32}$$

Therefore, the replication dynamic equation of the central government’s emergency dispatching strategy is:

(14) $$H\left(z\right)=\frac{dz}{dt}=z\left(U_{31}-\bar{U_{3}}\right)=z\left(1-z\right)\left[-\left(1-\varpi \right)C_{3}+R_{3}+x\theta L_{3}+y\theta W\right]$$

Further, the derivation of the replication dynamic equation of the central government’s emergency dispatching strategy is obtained as:

(15) $$H^{\prime }\left(z\right)=\left(1-2z\right)\left[-\left(1-\varpi \right)C_{3}+R_{3}+x\theta L_{3}+y\theta W\right]$$

To sum up, we have obtained the replication dynamic system of the cross-regional emergency cooperation game for natural disasters as follows:

(16) $$\left\{ \begin{array}{l} F\left(x\right)=x\left(1-x\right)\left[R_{1}-\left(1-\mu \right)M_{1}+\varpi P_{1}+z\left(1-\varpi \right)P_{1}+y\beta C_{1}-\left(1-y\alpha \right)\left(1-\eta \right)H_{1}+\left(y\alpha +z\theta -yz\alpha \theta \right)L_{1}\right]\\ G\left(y\right)=y\left(1-y\right)\left[R_{2}-\left(1-\mu \right)M_{2}+\varpi \left(E+P_{2}\right)+z\left(1-\varpi \right)\left(E+P_{2}\right)+x\beta C_{2}-\left(1-x\alpha \right)\left(1-\eta \right)H_{2}+x\alpha L_{2}\right]\\ H\left(z\right)=z\left(1-z\right)\left[-\left(1-\varpi \right)C_{3}+R_{3}+x\theta L_{3}+y\theta W\right] \end{array}\right.$$

## 4. Strategic Stability of All Game Subjects in Cross-Regional Emergency Cooperation for Natural Disasters

### 4.1. Analysis of the Strategic Stability of the Local Government

When F(x)=0 and $F^{\prime }\left(x\right)<0$ are satisfied, the probability of the local government’s emergency cooperation strategy is stable. Ordering F(x)=0, $y_{0}=-\frac{R_{1}-\left(1-\mu \right)M_{1}+\varpi P_{1}+z\left(1-\varpi \right)P_{1}-\left(1-\eta \right)H_{1}+z\theta L_{1}}{\alpha \left(1-z\theta \right)L_{1}+\alpha \left(1-\eta \right)H_{1}+\beta C_{1}}$ or $z_{0}=-\frac{R_{1}-\left(1-\mu \right)M_{1}+\varpi P_{1}+y\beta C_{1}-\left(1-y\alpha \right)\left(1-\eta \right)H_{1}+y\alpha L_{1}}{\theta \left(1-y\alpha \right)L_{1}+\left(1-\varpi \right)P_{1}}$ can be obtained by solving the equation.

**Figure 2:**
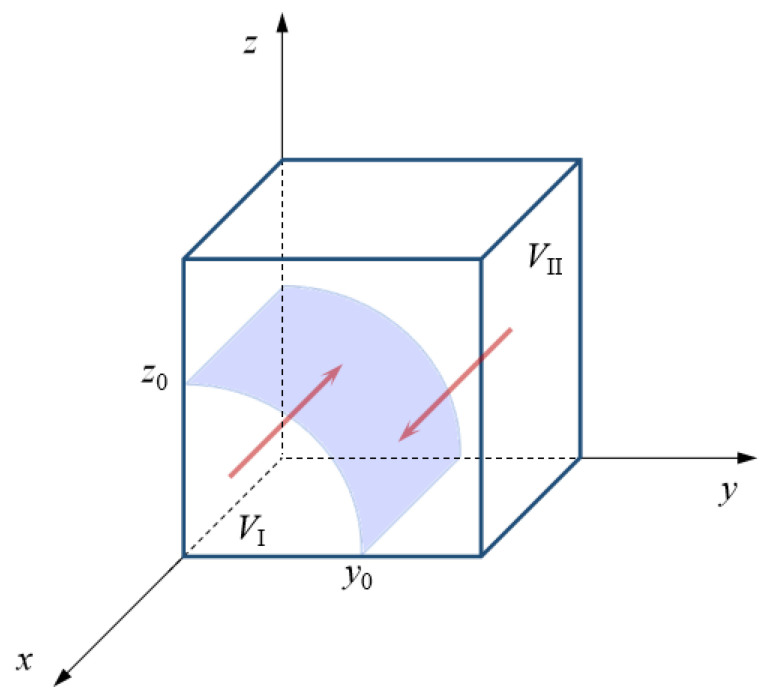
Evolutionary phase diagram of the local government’s strategy.

Further, when *z* > *z*_0_, both $F^{\prime }\left(x\right)|{}_{x=0}>0$ and $F^{\prime }\left(x\right)|{}_{x=1}<0$ are true. At this point, *x* = 1 (positive emergency cooperation) is the stable strategy of the local government. On the contrary, when *z* < *z*_0_, both $F^{\prime }\left(x\right)|{}_{x=0}<0$ and $F^{\prime }\left(x\right)|{}_{x=1}>0$ are true. Correspondingly, *x* = 0 (negative emergency cooperation) turns into the stable strategy of the local government. Thus, the evolutionary phase diagram of the local government’s emergency cooperation strategy can be obtained, as shown in Figure 2. The volume *V*_I_ of region I represents the probability that the local government chooses a negative emergency cooperation strategy. Similarly, the volume *V*_II_ of region II represents the probability that the local government chooses a positive emergency cooperation strategy.

According to the evolutionary phase diagram of the local government’s strategy, the critical value *z*_0_ of the central government’s emergency dispatching strategy is smaller, which means that the local government has a higher probability of choosing a positive emergency cooperation strategy. On the contrary, the larger the *z*_0_ is, the more the local government will adopt a negative emergency cooperation strategy. Specifically, when *z* > *z*_0_, the emergency cooperation strategy of the local government will converge to *x* = 1. The local government will avoid the responsibility penalty from the central government and will reduce the loss from natural disasters. On the contrary, when the central government chose the probability of a strong emergency dispatching strategy to be lower than a certain level (*z* < *z*_0_), the local governments realize that the emergency response gains and the mitigation of disaster losses from adopting a positive strategy are limited. Therefore, the probability of the local government’s positive emergency cooperation strategy will gradually converge to 0.

Similarly, when the critical value *y*_0_ of the neighboring government’s strategy decreases, the probability of the local government’s positive emergency cooperation strategy will increase. Otherwise, an increase in *y*_0_ indicates that the local government is more likely to adopt a negative emergency cooperation strategy. On the one hand, when the probability of the neighboring governments strategy is greater than the critical value *y*_0_, the positive emergency cooperation strategy of the local government will be the evolutionarily stable strategy. This depends on the matching targets of social responsibility, loss perception, and emergency rescue between the local and neighboring governments. Only by maintaining the consistency of actively responding to natural disasters can the emergency capital stock fully embrace its resource replenishment effect and improve the efficiency of emergency cooperation. This is more conducive to achieving the purpose of saving emergency costs and reducing natural disaster losses. At the same time, there will be no “free-riding” behavior in cross-regional emergency cooperation. The probability of the emergency cooperation strategy will tend to one. On the other hand, when the probability of the neighboring government’s positive emergency support strategy is low (*y* < *y*_0_), the local government realizes that it needs to pay more coordination, organization, and command costs in order to achieve orderly cooperation with the neighboring government. At the same time, the imbalance in emergency cooperation and the decentralization of emergency resources will not be able to reduce the local government’s perception of losses resulting from natural disasters. Thus, it will prompt the local government to choose a negative emergency cooperation strategy.

To sum up, the probability of a positive emergency cooperation strategy is directly proportional to the probability of a positive emergency support strategy and a strong emergency dispatching strategy.

### 4.2. Analysis of the Strategic Stability of the Neighboring Government

For the neighboring government, when G(y)=0 and $G^{\prime }\left(y\right)<0$, the probability of an emergency support strategy is stable. Ordering G(y)=0, $x_{0}=-\frac{R_{2}-\left(1-\mu \right)M_{2}+\varpi \left(E+P_{2}\right)+z\left(1-\varpi \right)\left(E+P_{2}\right)-\left(1-\eta \right)H_{2}}{\beta C_{2}+\alpha \left(1-\eta \right)H_{2}+\alpha L_{2}}$ or $z_{0}=-\frac{R_{2}-\left(1-\mu \right)M_{2}+\varpi \left(E+P_{2}\right)+x\beta C_{2}-\left(1-x\alpha \right)\left(1-\eta \right)H_{2}+x\alpha L_{2}}{\left(1-\varpi \right)\left(E+P_{2}\right)}$ can be calculated using Equation (9).

**Figure 3:**
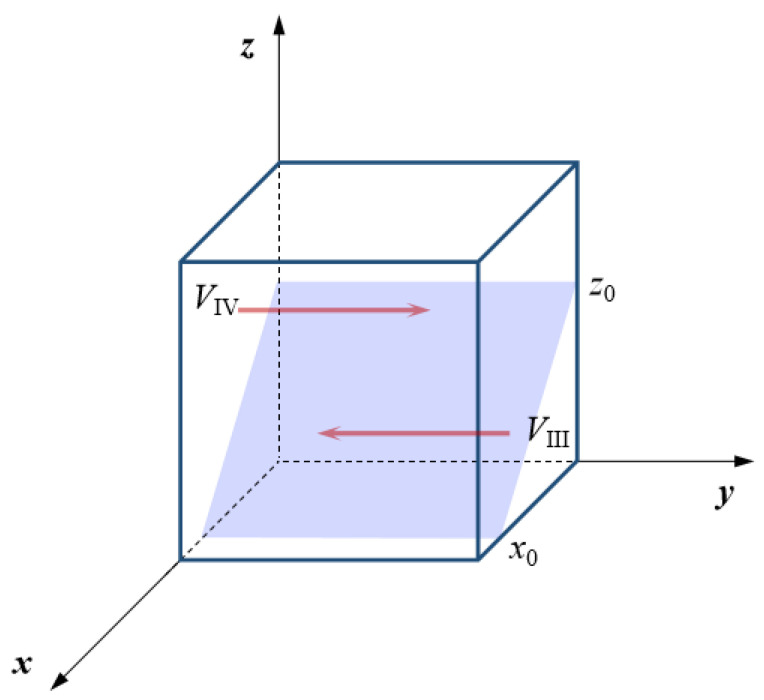
Evolutionary phase diagram of the neighboring government’s strategy.

Accordingly, when *x* > *x*_0_, both $G^{\prime }\left(y\right)|{}_{y=0}>0$ and $G^{\prime }\left(y\right)|{}_{y=1}<0$ are true. At this point, *y* = 1 (positive emergency support) is the stable strategy of the neighboring government. On the contrary, when *x* < *x*_0_, both $G^{\prime }\left(y\right)|{}_{y=0}<0$ and $G^{\prime }\left(y\right)|{}_{y=1}>0$ are true. Correspondingly, *y* = 0 (negative emergency support) turns into the stable strategy of the neighboring government. Furthermore, the evolutionary phase diagram of the neighboring government’s emergency support strategy can be drawn. As can be seen from Figure 3, the volume *V*_III_ of region III represents the probability that the neighboring government chooses a negative emergency support strategy. Similarly, the volume *V*_IV_ of region IV represents the probability that the neighboring government chooses a positive emergency support strategy.

According to the evolutionary phase diagram of the neighboring government’s strategy, it is more likely that the neighboring government will choose a positive emergency support strategy when the critical value *x*_0_ is smaller. Correspondingly, the larger the critical value *x*_0_ is, the greater the probability of choosing a negative emergency support strategy. Similarly, when the critical value *z*_0_ of a strong emergency dispatching strategy is small, the probability of a positive emergency support strategy is high. At the same time, with an increase in *z*_0_, the probability of a positive emergency cooperation strategy will gradually decrease.

From the perspective of the local government, when the probability of a positive emergency cooperation strategy is greater than the critical value *x*_0_, a positive emergency support is the stable strategy of the neighboring government. When the local government and the neighboring government choose a positive emergency strategy at the same time, the cost of the emergency, the dissipative cost of emergency cooperation, and perceived losses from natural disasters will be effectively reduced. In turn, the neighboring government’s emergency support strategy will converge to *y* = 1. At the same time, this also reflects that the neighboring government with a good foundation of emergency cooperation will not choose to deal with natural disasters through the local government alone. On the contrary, when the probability of a positive emergency cooperation strategy is lower than a certain level (*x* < *x*_0_), an increase in the implementation cost will reduce the enthusiasm and initiative of the neighboring government to participate in cross-regional emergency cooperation. Moreover, the neighboring government’s positive strategy cannot avoid a decline in the cooperation level of resources, equipment, and information between the local and neighboring governments, and its game strategy will eventually stabilize at *y* = 0.

From the perspective of the central government, when the probability of a strong emergency dispatching strategy is greater than *z*_0_, the central government’s emergency dispatching will be at a high level. In order to reduce the risk of being punished by the central government, the neighboring government will have a higher tendency to choose a positive emergency support strategy. At this time, the neighboring government will also obtain compensation income from the central government. On the contrary, when the probability of a strong emergency dispatching strategy is low (*z* < *z*_0_), the neighboring government without vertical administrative constraints will weaken the level of emergency support because of the lack of incentives for emergency operations. The emergency support strategy will converge to *y* = 0.

Therefore, the probability of a positive emergency support strategy is directly proportional to an increase in the probability of a positive emergency cooperation strategy and a strong emergency dispatching strategy.

### 4.3. Analysis of the Strategic Stability of the Central Government

For the central government, when H(z)=0 and $H^{\prime }\left(z\right)<0$, the probability of an emergency dispatching strategy is stable. Ordering H(z)=0, $x_{0}=-\frac{-\left(1-\varpi \right)C_{3}+R_{3}+y\theta W}{\theta L_{3}}$ or $y_{0}=-\frac{-\left(1-\varpi \right)C_{3}+R_{3}+x\theta L_{3}}{\theta W}$ can be calculated using Equation (14).

**Figure 4:**
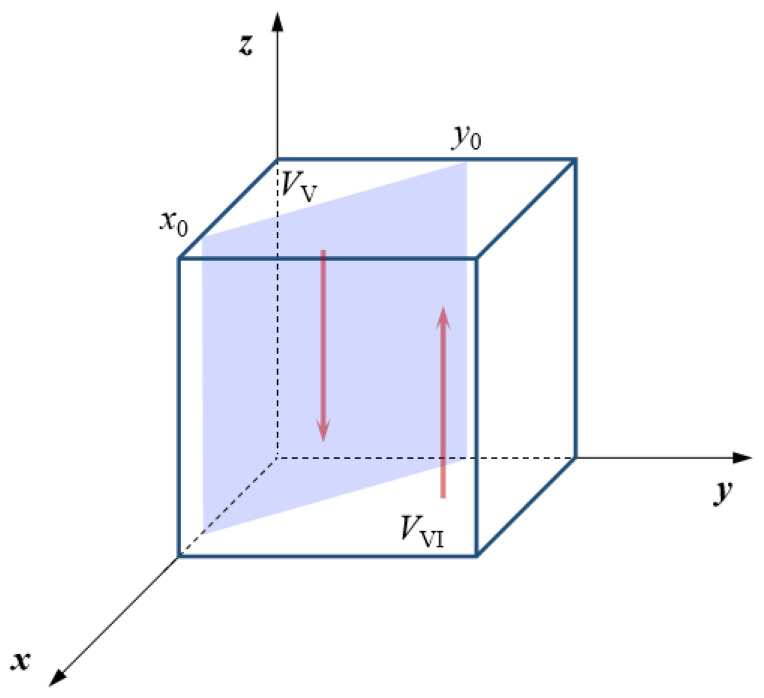
Evolutionary phase diagram of the central government’s strategy.

Accordingly, when *x* > *x*_0_, both $H^{\prime }\left(z\right)|{}_{z=0}>0$ and $H^{\prime }\left(z\right)|{}_{z=1}<0$ are true. At this point, *z* = 1 (strong emergency dispatching) is the stable strategy of the central government. On the contrary, when *x* < *x*_0_, both $H^{\prime }\left(z\right)|{}_{z=0}<0$ and $H^{\prime }\left(z\right)|{}_{z=1}>0$ are true. Correspondingly, *z* = 0 (weak emergency dispatching) turns into the stable strategy of the central government. Furthermore, the evolutionary phase diagram of the central government’s emergency dispatching strategy can be obtained, as shown in Figure 4. The volume *V*_v_ of region V represents the probability that the central government chooses a weak emergency dispatching strategy. Similarly, the volume *V*_VI_ of region VI represents the probability that the central government chooses a strong emergency dispatching strategy.

According to the evolutionary phase diagram of the central government’s emergency dispatching strategy, the smaller the critical value of the emergency cooperation strategy’s probability *x*_0_ is, the higher the probability of the central government’s strong emergency dispatching strategy is. On the contrary, with the improvement of *x*_0_, the central government will choose a weak emergency dispatching strategy. In a similar way, when the critical value of the emergency support strategy’s probability *y*_0_ is at a low level, the central government will have a high probability of choosing a strong emergency dispatching strategy. Correspondingly, the probability of a strong emergency dispatching strategy will decrease according to how great *y*_0_ is.

From the perspective of the local government, when the probability of a positive emergency cooperation strategy is greater than the critical value *x*_0_, the *θ* formed by the central government and the local governments will be at a higher level and the central government’s perception of losses caused by natural disasters will be reduced. At this time, the central government can gain more benefits from a strong emergency dispatching strategy. Otherwise, when the probability of a positive emergency cooperation strategy is low (*x* < *x*_0_), it is more difficult and complicated for the central government to dispatch the emergency response, which further increases the emergency cost incurred by the central government. Therefore, the probability of a strong emergency dispatching strategy will be reduced to some extent. From the perspective of the neighboring government, when the probability of a positive emergency support strategy is higher than a certain level (*y* > *y*_0_), the central government will gain more perceived emergency benefits under a strong emergency scheduling strategy. Therefore, the central government will converge to *z* = 1. On the contrary, when the probability of a positive emergency support strategy is less than the critical value *y*_0_, the central government will choose to weaken the intensity of the emergency dispatching, to reduce the cost of coordination and communication, force deployment, and material dispatching.

To sum up, with an increase in the probability of a positive emergency cooperation strategy and a positive emergency support strategy, the probability of a strong emergency dispatching strategy will also gradually increase.

## 5. Simulation Analysis of Cross-Regional Emergency Cooperation Game for Natural Disasters

In order to clearly and intuitively describe the dynamic evolution behavior of the local government, the neighboring government, and the central agency in the cross-regional emergency cooperation for natural disasters, a numerical simulation analysis was used to analyze the influence mechanism of variable parameter changes on the game subjects’ strategy evolution. Due to the diversity of research data and the abstractness of hypothetical variables, in this study, we use the Delphi method to organize the field demonstration and questionnaire scoring of experts. On this basis, the initial values of simulation parameters in the game model of cross-regional emergency cooperation for natural disasters were given in combination with the statistical data from the China Statistical Yearbook and the actual situation of China’s emergency management agencies. Assume that *L*_1_ = 10, *H*_1_ = 15, *C*_1_ = 10, *P*_1_ = 12, *M*_1_ = 15, *R*_1_ = 10, *L*_2_ = 10, *C*_2_ = 10, *E* = 10, *P*_2_ = 10, *H*_2_ = 10, *R*_2_ = 10, *M*_2_ = 15, *C*_3_ = 12, *R*_3_ = 10, *W* = 10, *L*_3_ = 10, and the initial values of the cooperation level parameters *θ*, *α*, *μ*, *η*, *β,* and *ω* are set to 0.1. In addition, the initial probability of each game subject’s strategy in the cross-regional emergency cooperation for natural disasters is set as 0.5.

### 5.1. The Effect of θ on the Evolution of the Game System

**Figure 5:**
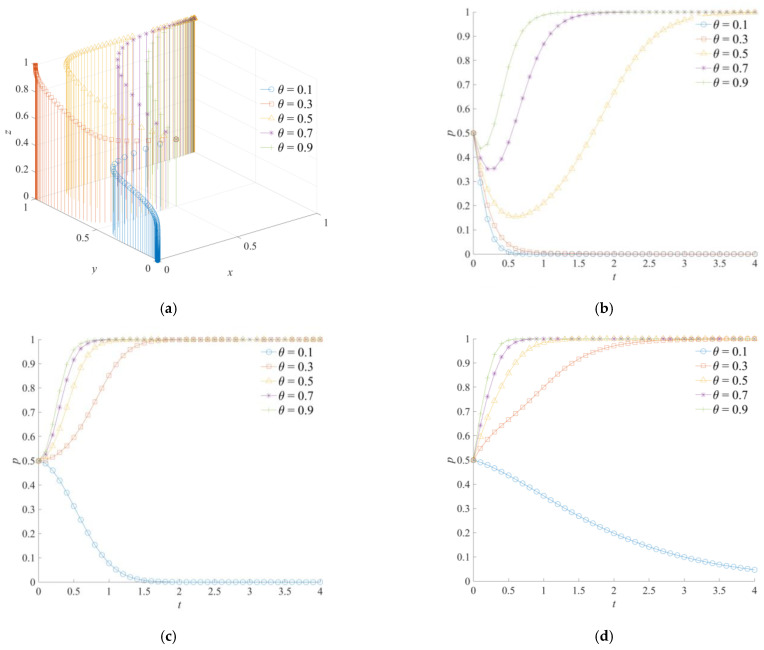
Evolution trajectory of the game system under different effective coefficients of national–local government emergency dispatching: (**a**) The influence of *θ* on the evolution of the game system; (**b**) the influence of *θ* on the evolution of the local government; (**c**) the influence of *θ* on the evolution of the neighboring government; (**d**) the influence of *θ* on the evolution of the central government.

Figure 5 shows the game system’s evolution trajectory of the cross-regional emergency cooperation for natural disasters under the different values of *θ*. When the effective coefficient of the national-local government emergency dispatching *θ* is at a low level, the local, neighboring, and central governments will evolve to negative emergency cooperation, negative emergency support, and a weak emergency dispatching strategy, respectively. Meanwhile, a higher θ indicates that the central government’s emergent command is more direct and effective. With an increase in *θ*, the neighboring and central governments will, first, change to positive emergency cooperation and a strong emergency dispatching strategy, respectively. At this point, the system’s stable-state strategy of the cross-regional emergency cooperation for natural disasters is (0,1,1). Furthermore, when *θ* = 0.5, *θ* = 0.7, or *θ* = 0.9, positive emergency cooperation, positive emergency support, and strong emergency dispatching will become the strategy combination of the game system. At the same time, with an increase in *θ*, the convergence speed of the game subject to the stable strategy accelerates. As shown in Figure 5a, the local government will reduce the perception of various losses caused by natural disasters with a higher *θ*, so the local government will tend to choose a positive emergency cooperation strategy. However, although the positive emergency cooperation strategy is the final strategy of the local government, the local government will take the lead in converging to a negative emergency cooperation strategy in the initial stage of evolution. As shown in Figure 5c, an increase in *θ* has a greater impact on the strategic change of the central government than that of the local government. Under the incentive of high efficiency of emergency operations and a reduction in natural disaster losses, the central government will evolve to a strong emergency dispatching strategy more quickly. In addition, although the θ has no direct influence on the strategy choice of the neighboring government, with the change in the strategies of the local and central governments, the neighboring governments with the same emergency goals will also choose a positive emergency support strategy in order to respond.

### 5.2. The Effect of α on the Evolution of the Game System

**Figure 6:**
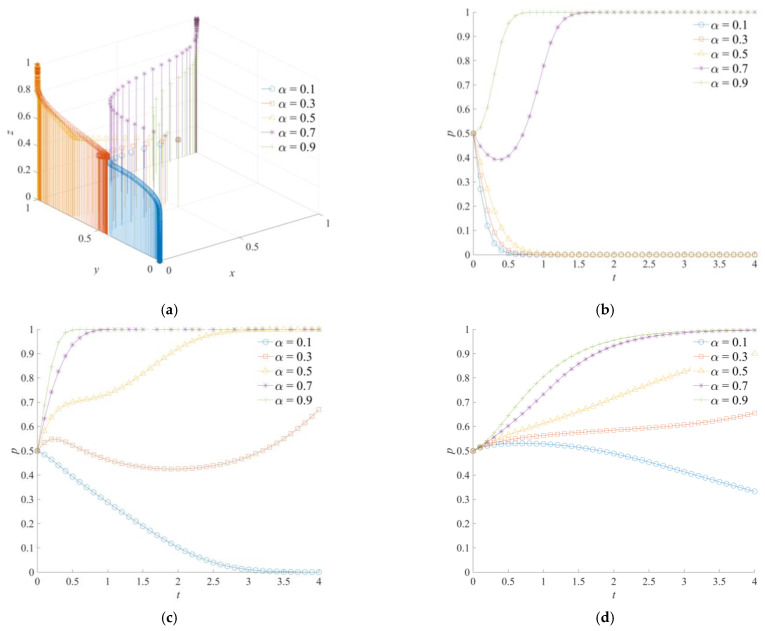
Evolution trajectory of the game system under different efficiency coefficients of emergency rescue cooperation: (**a**) The influence of *α* on the evolution of the game system; (**b**) the influence of *α* on the evolution of the local government; (**c**) the influence of *α* on the evolution of the neighboring government; (**d**) the influence of *α* on the evolution of the central government.

The game system’s evolutionary trajectory of the cross-regional emergency cooperation for natural disasters was obtained through a simulation analysis, as shown in Figure 6. Among them, the efficiency coefficient of emergency rescue cooperation *α* is set as 0.1, 0.3, 0.5, 0.7 and 0.9, respectively. The efficiency coefficient of emergency rescue cooperation is a variable that can describe the implementation effect of the local and neighboring governments’ emergency operations. As shown in Figure 6a, (0,0,0), (0,1,1), and (1,1,1) are the stable combinations that are formed by the game system’s strategy of the cross-regional emergency cooperation for natural disasters. In Figure 6b, the dissipative cost of emergency cooperation of the local government will be reduced when the efficiency coefficient of emergency rescue cooperation becomes larger. At the same time, the possibility of mitigating the loss of natural disasters and avoiding secondary disasters is greater. Therefore, when *α* = 0.7 or *α* = 0.9, positive emergency cooperation is the evolutionarily stable strategy of the local government. In addition, the convergence rate of the local government to the stable strategy is significantly faster than that of the neighboring and central governments. This shows that the local government plays a major role in promoting the overall evolution of the game system. As shown in Figure 6c, when the value of *α* is low, the neighboring government is more inclined to choose a negative emergency support strategy. With an increase of *α*, the neighboring government has a temporary wait-and-see strategy, and finally chooses a positive emergency support strategy as the evolution time increases. As can be seen from Figure 6d, the strategic choice of the central government is adapted to the changes in the implementation effects of emergency operations between the local and neighboring governments. The higher a is, the greater the expected benefits of the central government for cross-regional emergency cooperation will be, and the more positive the central government tends to choose a strong emergency dispatching strategy. However, the evolution of the central government to the stable strategy is slower than that of the local and neighboring governments.

### 5.3. The Effect of μ on the Evolution of the Game System

**Figure 7:**
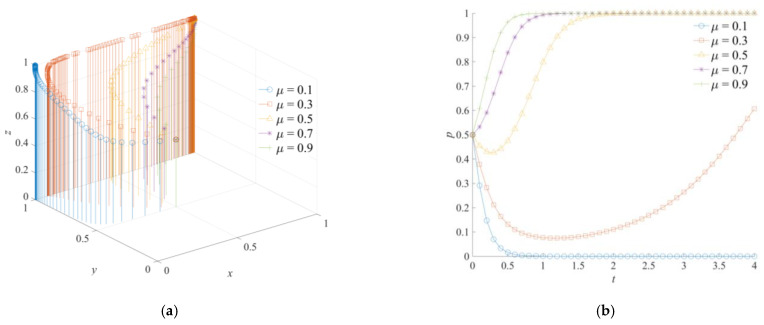

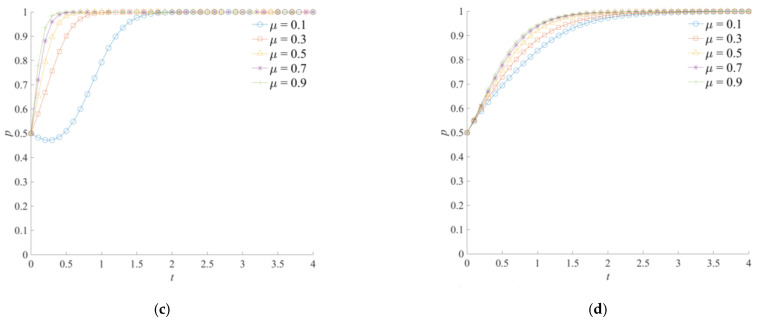
Evolution trajectory of the game system under different response coefficients of social emergency forces: (**a**) The influence of *μ* on the evolution of the game system; (**b**) the influence of *μ* on the evolution of the local government; (**c**) the influence of *μ* on the evolution of the neighboring government; (**d**) the influence of *μ* on the evolution of the central government.

The evolution results of the cross-regional emergency cooperation game system are shown in Figure 7. Therefore, the response coefficients of social emergency forces *μ* are set as 0.1, 0.3, 0.5, 0.7, and 0.9. Social emergency forces are important supplements and auxiliary forces of the cross-regional emergency cooperation for natural disasters. More specifically, social emergency forces are mainly responsible for assisting government rescue forces and coordinating with emergency support agencies to carry out logistical support tasks in cross-regional emergency cooperation for natural disasters. When the response coefficient of social emergency forces *μ* = 0.1, the game system will converge to (0,1,1). With an increase in *μ*, each game subject will effectively save the costs of emergency and integration of social emergency forces. Additionally, the phenomenon of negative emergency and inaction of the game subjects will not be formed. The game system of cross-regional emergency cooperation for natural disasters gradually evolved into a strategy combination of active emergency cooperation, active emergency support, and strong emergency dispatching. By comparing Figure 7a–c, it can be seen that social emergency forces have a more direct and significant impact on the strategic choice of the local government. The response of social emergency forces and the local government’s emergency operations show mutual promotion. The supporting effect of social emergency forces is more prominent. At the same time, the improvement of *μ* will accelerate the evolution of the neighboring government to a positive support strategy and the central government to a strong dispatching strategy. However, due to the high coordination cost and the lack of an early emergency response basis, the social emergency forces mainly reflect the external assistance effect of the neighboring and central governments, but have limited influence on the strategic choices of the neighboring and central governments.

### 5.4. The Effect of η on the Evolution of the Game System

**Figure 8:**
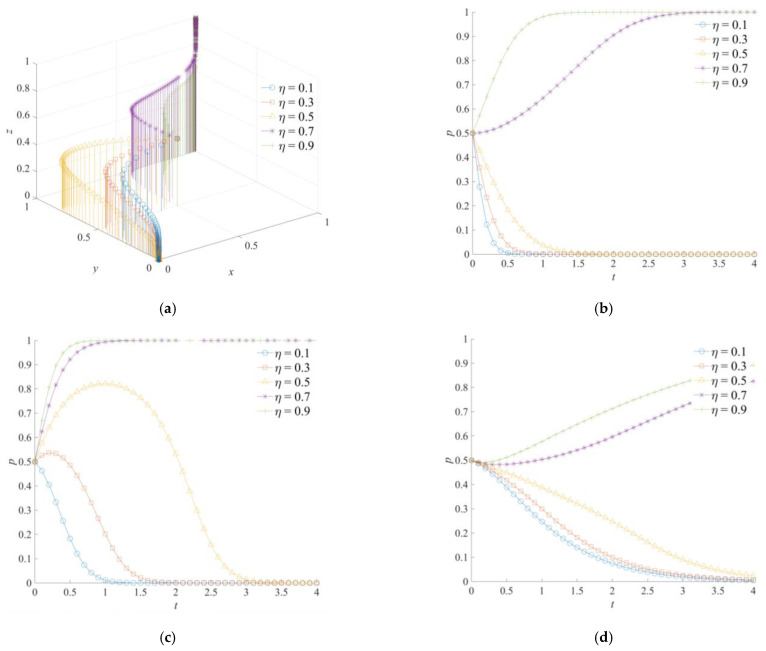
Evolution trajectory of the game system under different matching degrees of cross-regional emergency rescue: (**a**) The influence of *η* on the evolution of the game system; (**b**) the influence of *η* on the evolution of the local government; (**c**) the influence of *η* on the evolution of the neighboring government; (**d**) the influence of *η* on the evolution of the central government.

Figure 8 shows the evolution trajectory of the game system of the cross-regional emergency cooperation for natural disasters under different *η* levels. When the matching degree of cross-regional emergency rescue *η* is at a low level, the game subjects will evolve to negative emergency cooperation, negative emergency support, and weak emergency dispatching, respectively. Increasing the matching degree of cross-regional emergency rescue will promote the positive emergency cooperation of the local government, the positive emergency support of the neighboring government, and the strong emergency dispatching of the central government. Eventually, the combination of (1,1,1) strategies will be formed. When the *η* level is set higher, the local and neighboring governments will have strong adaptability in emergency task allocation and emergency information exchange, which can effectively avoid problems such as poor information communication and blocked docking channels. The additional costs of emergency dissipating “entropy increase” by the local and neighboring governments also decreases simultaneously. In Figure 8b, when *η* = 0.7 or *η* = 0.9, the local government will converge to *x* = 1. Meanwhile, in Figure 8c, when *η* is set as 0.5, 0.7, and 0.9, the neighboring government will converge to *y* = 1. This indicates that the matching degree of cross-regional emergency rescue has a more significant impact on the neighboring government strategy. In addition, as shown in Figure 8d, the central government gradually converges to *z* = 1 under the conditions of a high matching degree *η*, driven by the change in the local and neighboring governments’ strategies. It can be seen that the central government is not sensitive to a change in *η*, so the evolution of the stable strategy is slow. In addition, in the game system of the cross-regional emergency cooperation for natural disasters, the strategy selection of each game subject has an evolutionary trend of coordinated development and following change.

### 5.5. The Effect of β on the Evolution of the Game System

**Figure 9:**
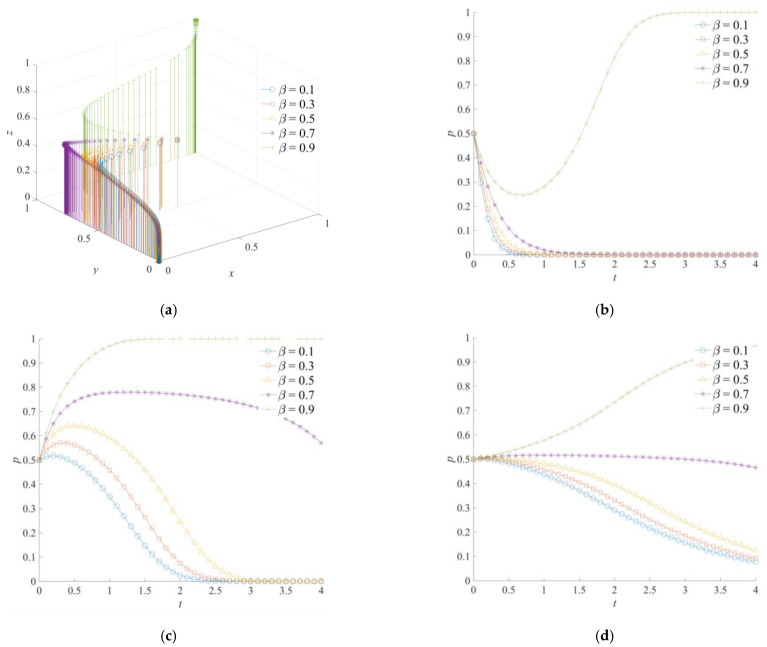
Evolution trajectory of the game system under different coverage effect coefficients of emergency capital: (**a**) The influence of *β* on the evolution of the game system; (**b**) the influence of *β* on the evolution of the local government; (**c**) the influence of *β* on the evolution of the neighboring government; (**d**) the influence of *β* on the evolution of the central government.

The coverage effect coefficient of emergency capital *β* is set as 0.1, 0.3, 0.5, 0.7, and 0.9. The game system’s evolutionary track of the cross-regional emergency cooperation for natural disasters is shown in Figure 9; (0,0,0) and (1,1,1) are the possible evolutionary results of the game system. When *β* = 0.9, positive emergency cooperation, positive emergency support, and strong emergency dispatching are the evolutionarily stable strategies of the system. With a decrease in *β*, the difference in emergency costs paid between a positive strategy and a negative strategy by the local and neighboring governments is less than 0, and the difference in emergency costs paid between a strong strategy and a weak strategy of the central government is also less than 0. Therefore, the game system will converge to (0,0,0).

According to Figure 9b,c, although the local and neighboring governments have the same strategy direction in the process of improving *β*, the evolution processes of the two strategies are different. When the coverage effect coefficient of emergency capital is at a low level, the evolution of the local government to a negative emergency cooperation strategy is more direct. Moreover, as the emergency capital stock of the local government becomes smaller, its convergence speed to a negative emergency cooperation strategy becomes faster. Meanwhile, with the advance in evolution time, the neighboring government’s strategy selection presents an evolutionary trend of first choosing a positive strategy and then turning to a negative strategy. Accordingly, when the emergency capital stock between the territorial and the neighboring governments is insufficient, the neighboring government will positively respond to emergency operations due to functional requirements and central command in the early stages of evolution. However, at the end of the evolution, the neighboring government will change to a negative support strategy because of the high support costs.

For the central government, its strategy choice is mainly affected by the strategic orientations of the local and neighboring governments. When the local and neighboring governments choose a positive emergency strategy, the central government will also choose a strong emergency dispatching strategy. On the contrary, when the local and neighboring governments choose a negative emergency strategy, a weak emergency dispatching strategy will become the final choice of the central government. Therefore, the strategic choices of the local, neighboring, and central governments have the features of consistency and following.

### 5.6. The Effect of ω on the Evolution of the Game System

**Figure 10:**
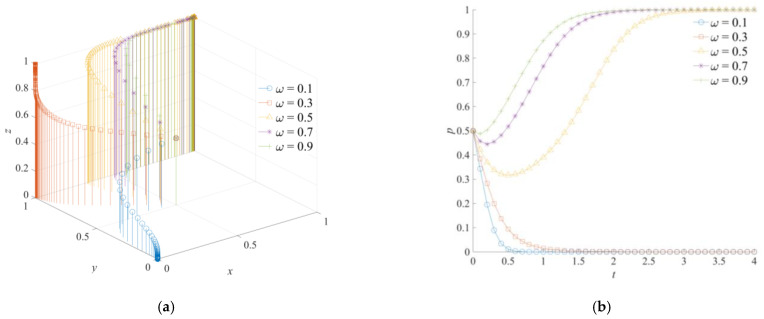

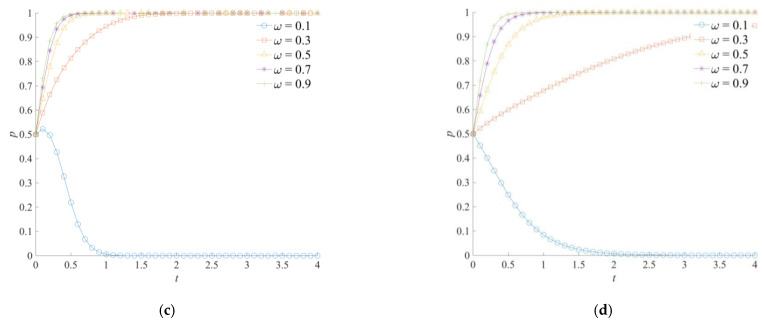
Evolution trajectory of the game system under different emergency dispatching degrees of the central government: (**a**) The influence of *ω* on the evolution of the game system; (**b**) the influence of *ω* on the evolution of the local government; (**c**) the influence of *ω* on the evolution of the neighboring government; (**d**) the influence of *ω* on the evolution of the central government.

The emergency dispatching degree of the central government *ω* is set as 0.1, 0.3, 0.5, 0.7, and 0.9. The game system’s simulation evolution results of the cross-regional emergency cooperation for natural disasters are shown in Figure 10. The emergency dispatching degree of the central government *ω* is a parameter that could describe the implementation intensity of the central government’s participation in natural disaster emergency operations. As shown in Figure 10a, with an increase in the central government’s emergency dispatching intensity, the game system’s steady-state strategy of the cross-regional emergency cooperation for natural disasters forms a development path from (0,0,0) to (0,1,1), and then to (1,1,1). In Figure 10b, an increase in the central government’s emergency dispatching intensity will enhance the local government’s loss perception of the administrative accountability punishment under a negative emergency strategy, and therefore the higher intensity of the central government’s emergency dispatching will weaken the formation trend of a negative emergency cooperation strategy by the local government and promote the formation trend of a positive emergency cooperation strategy. In Figure 10c, as the neighboring government gains more reward for emergency compensation and less loss for administrative accountability under a positive emergency support strategy, with an increase in *ω*, the strategic choice of the neighboring government will change from a negative emergency support to a positive emergency support strategy. At the same time, the evolution speed of the neighboring government’s choice to a positive emergency strategy will become faster. In Figure 10d, when *ω* is at a high level, the relative net payment of the central government choosing a strong emergency dispatching strategy is positive, so the central government will choose a strong emergency dispatching strategy. In general, a higher emergency dispatching intensity will promote the subjects of the cross-regional emergency cooperation to choose a positive emergency strategy. Meanwhile, the neighboring and the governments will react more sensitively to the changes in the parameters of *ω*, and their strategies will change more quickly.

## 6. Discussion

The response and disposal of natural disasters is an interactive process of multi-agent coordination and cooperation. As natural disasters have prominent cross-regional characteristics, the response and disposal of natural disasters involve different regions, different levels of government, and relevant departments. The key to the response and mitigation of natural disasters is the efficiency and effectiveness of emergency cooperation. The game model of cross-regional emergency cooperation for natural disasters, based on the game subjects of the local, neighboring, and central governments, describes this problem effectively. The practical path to improve the performance of a natural disaster emergency response is also given.

According to the simulation results of the game system, the evolution of the cross-regional emergency cooperation for natural disasters, the regional, neighboring, and central governments will evolve in opposite strategic directions over a certain period of time. However, because each game subject has the same emergency management goal when dealing with natural disasters, the local, neighboring, and central governments are the driving forces for the evolution of each other’s strategies. The mutual promotion of positive emergency strategies and the coordinated deployment of the emergency operations are the basic connotations of the strategy selection of the game subjects.

Overall, the evolution and development of the cross-regional emergency cooperation game system shows the consistency of the game subjects’ strategies. At the same time, in order to clarify the interaction mechanism of each subject in the cross-regional emergency cooperation for natural disaster, in this study, we introduce six parameter variables to measure the attribute characteristics of the game subjects, which were based on social capital theory, dissipative structure theory, and signaling theory. The six parameter variables are: the coverage effect coefficient of emergency capital, the efficiency coefficient of emergency rescue cooperation, the response coefficient of social emergency forces, the effective coefficient of national–local government emergency dispatching, the emergency dispatching degree of the central government, and the matching degree of cross-regional emergency rescue. Furthermore, the influence mechanisms of *θ*, *α*, *μ*, *η*, *β,* and *ω* on the game system are revealed.

In this study, the basic assumptions about cross-regional emergency cooperation for natural disasters were derived from objective descriptions from the Chinese government’s emergency management practice. The study results have typical Chinese situation characteristics. However, this study will still have some theoretical and practical value for governments around the world to deal with natural disasters and to improve the cooperation mechanism of emergency organizations. From the perspective of multi-level government, in this study, we emphatically analyze the interactions among the local, the neighboring, and central governments. However, in the practice of the cross-regional emergency cooperation for natural disasters, there are still organizational interaction forms, such as cross-regional reinforcement of different administrative departments and cross-regional participation of social emergency forces. Exploring the emergency cooperation between different government departments at the same level and the emergency interaction between government rescue forces and social emergency forces are also the key topics of this study and need further detailed discussion and in-depth study.

In addition, this study is an important attempt to describe the cooperative relationship between emergency organizations in natural disasters. As compared with the existing studies, this study focuses on the cross-regional coordination, cross-regional command, and cross-regional support attributes of the governments at all levels in the process of coping with natural disasters. At the same time, social capital theory, dissipative structure theory, and signaling theory are introduced into the game model hypothesis of the cross-regional emergency cooperation for natural disasters. To some extent, the perspective and scope of the research on the organizational relationships of emergency management were broadened. However, this study is more of a basic description of different interaction relationships, such as the basis of emergency cooperation, redundancy in emergency cooperation, and information transmission in emergency cooperation. A preliminary conclusion, consistent with the practice of emergency cooperation, is obtained, but still needs to be further explored for a specific relationship in future study. At the same time, the development of social capital theory, dissipative structure theory, and signaling theory in the study of emergency cooperation must be constantly promoted.

## 7. Conclusions

In order to clearly describe the organizational interactions in cross-regional emergency cooperation for natural disasters, a three-agent evolutionary game model of local, neighboring, and central governments is constructed. Meanwhile, the strategy stability and evolution direction of each game subject are analyzed. Furthermore, the decision-making process and development trajectory of cross-regional emergency cooperation for natural disasters are discussed through a numerical simulation. The main research conclusions are as follows:

(1) During a natural disaster emergency response, the local, neighboring, and central governments hope to improve the efficiency of emergency cooperation to reduce losses. Therefore, when each government chooses an active emergency strategy, it promotes the other two governments to choose a positive emergency strategy. This indicates that, in the game model of cross-regional emergency cooperation for natural disasters, there is no “free rider” phenomenon between governments.
(2) Strengthening the administrative strength of the central government in emergency command and emergency dispatching will promote the local and neighboring governments to choose a positive emergency cooperation strategy and improve the efficiency of natural disaster emergency cooperation. At the same time, improving the effectiveness of the central government’s emergency command and the local government’s response will enhance the level of interaction between central–local governments, and encourage governments to fulfill their respective duties.
(3) In order to improve the efficiency of emergency response and emergency management between the local and neighboring governments, it can be carried out from two aspects: (1) enhancing the resource sharing level and (2) deepening the tacit understanding degree and function matching degree. Enhancing the level of resource sharing and tacit understanding will improve the integrity of emergency cooperation between the local government and the neighboring government.
(4) The simulation results show that cultivating and accumulating emergency capital stock between the local and neighboring governments is an effective measure for reducing emergency costs. Cross-regional emergency cooperation, cross-regional emergency agreement and plan preparation, and natural disaster forecast consultation are the main sources of accumulation of emergency capital stock.
(5) The participation of social emergency forces is an important supplement to governments for natural disaster emergency response, which will reduce the emergency costs of governments. Meanwhile, the participation of social emergency forces will affect the degree of supplement for governments’ emergency responses. Therefore, governments at all levels should strengthen incentives and support for social emergency forces and guide social emergency forces to actively and orderly participate in natural disaster emergency responses.

### Implications

In order to improve the performance of cross-regional emergency cooperation for natural disasters, to complete the institution of the cross-regional emergency cooperation for natural disasters, and to optimize the organizational structure of the government in response to natural disasters, combined with the above research conclusions, we propose the following countermeasures that are in line with the law of natural disaster prevention and the situation of government emergency management:

(1) Governments need to improve the mechanism of cross-regional emergency cooperation for natural disasters. First, neighboring governments should sign cross-regional emergency cooperation agreements and establish cross-regional emergency consultation mechanisms. Then, each government needs to analyze potential risks, problems of preference, and development trends in its respective regions in order to propose joint preventive response measures and to ensure emergency preparedness for natural disasters. Second, it is necessary to improve the joint preparation of emergency plans for natural disasters. In addition, the national natural disaster emergency plan and local natural disaster emergency plan should be further connected and coordinated. The specific responsibilities of governments and functional departments in cross-regional emergency cooperation should be defined. Third, the government should actively carry out joint exercises of trans-regional emergency responses for natural disasters to run the linkage mechanism. The level of emergency preparedness and emergency combat capability of the government and functional departments could be enhanced significantly.
(2) Government should optimize the cross-regional emergency command structure for natural disasters. First, an emergency command system with clear instructions from higher levels and smooth feedback from lower levels should be built, in order to form a flattened emergency command structure in which the field headquarters and the national central headquarters respond to each other. Furthermore, the synergistic efficiency of cross-regional emergency cooperation for natural disasters can be improved. Second, the government rescue and social emergency forces should be integrated in order to build a multi-level and integrated natural disaster emergency force system with government rescue forces as the core and various forms of emergency forces as the support. The organizational foundation for coordination of emergency operations and effective docking of emergency needs will gradually be consolidated. Third, the overall implementation of cross-regional emergency support and the overall scheduling of emergency resources should be realized. Governments need to establish the whole-process material allocation mode of accompanying support and act as a territorial guarantee. Then, the sharing level of emergency equipment will be improved, and the rapid, accurate, and efficient delivery of emergency materials will be realized.
(3) Governments need to improve the scientific and technological level of natural disaster emergency cooperation. On the one hand, a natural disaster emergency command and dispatch system should be built to strengthen the organizational coordination performance of governments and functional departments. At the same time, it is necessary to expand the information access port of the system. Then, timely notification of emergency information, such as disaster situation notification and rescue demand, can be realized, and the interactive level of natural disaster information will be further enhanced. Furthermore, the establishment of an integrated service platform for social emergency forces should be accelerated. Governments should understand the personnel size and equipment structure of the social emergency force in a timely manner in order to further strengthen daily management and operational guidance. In addition, the relevant functional departments should encourage and guide the social emergency forces in order to orderly participate in the cross-regional emergency operations for natural disasters.
(4) The applicability of this study still has some limitations. This is an exploratory study on the cross-regional emergency cooperative relationship for natural disasters. While we have preliminarily analyzed the factors that affect the strategy choice of emergency organization, the mechanism of influencing factors has not been explained in detail from the dynamic, systematic, and root levels. The model still needs to be adjusted and optimized according to the complicated internal and external environment in natural disaster emergency. Furthermore, we adopted a numerical simulation method to analyze the influence of different parameters on the game system’s evolution. As a next step, the actual data and case simulations need to be combined in order to carry out an in-depth discussion and analysis.

## Figures and Tables

**Table 1:** Definition of parameters.

Symbol	Definition	Ranges
*C_i_*	Emergency costs for different governments’ responses to natural disasters.	*C_i_* > 0, *i* = 1, 2, 3
*H*_1_	Dissipative costs of cross-regional emergency cooperation of the local government.	*H*_1_ > 0
*H*_2_	Dissipative costs of cross-regional emergency cooperation of the neighboring government.	*H*_2_ > 0
*P*_1_	The central government’s penalties of accountability for negative cooperation of the local government.	*P*_1_ > 0
*P*_2_	The central government’s penalties of accountability for negative cooperation of the neighboring government.	*P*_2_ > 0
*L_i_*	The sum of different governments’ perceptions of losses incurred by natural disasters.	*L_i_* > 0, *i* = 1, 2, 3
*R_i_*	Social reputation benefits of emergency actions by different governments.	*R_i_* > 0, *i* = 1, 2, 3
*M*_1_	Management costs paid by the local government to integrate social emergency forces.	*M*_1_ > 0
*M*_2_	Management costs paid by neighboring governments to integrate social emergency forces.	*M*_2_ > 0
*E*	Compensation and subsidies from the central government to neighboring governments for emergency response operations.	*E* > 0
*W*	The benefit perception of the central government formed by the reduction of natural disaster losses.	*W* > 0
*β*	The coverage effect coefficient of emergency capital.	0 ≤ *β* < 1
*α*	The efficiency coefficient of emergency rescue cooperation.	0 ≤ *α* < 1
*μ*	The response coefficient of social emergency forces.	0 ≤ *μ* < 1
*θ*	The effective coefficient of national-local government emergency dispatching.	0 ≤ *θ* < 1
*ω*	The emergency dispatching degree of the central government.	0 ≤ *ω* ≤ 1
*η*	The matching degree of cross-regional emergency rescue.	0 ≤ *η* < 1

**Table 2:** The payment matrix under the central government’s strong emergency dispatching strategy.

				The Central Government
				Strong Emergency Dispatching
The local government	Positive emergency cooperation	The neighboring government	Positive emergency support	$-\left(1-\beta \right)C_{1}-\left(1-\eta \right)\left(1-\alpha \right)H_{1}-\left(1-\theta \right)\left(1-\alpha \right)L_{1}+R_{1}-\left(1-\mu \right)M_{1}$, $-\left(1-\beta \right)C_{2}-\left(1-\eta \right)\left(1-\alpha \right)H_{2}-\left(1-\alpha \right)L_{2}+R_{2}-\left(1-\mu \right)M_{2}+E$, $-C_{3}-\left(1-\theta \right)L_{3}+R_{3}+\theta W$
			Negative emergency support	$-C_{1}-\left(1-\eta \right)H_{1}-\left(1-\theta \right)L_{1}+R_{1}-\left(1-\mu \right)M_{1}$, $-C_{2}-L_{2}-P_{2}$, $-C_{3}-\left(1-\theta \right)L_{3}+R_{3}$
	Negative emergency cooperation		Positive emergency support	$-C_{1}-L_{1}-P_{1}$, $-C_{2}-\left(1-\eta \right)H_{2}-L_{2}+R_{2}-\left(1-\mu \right)M_{2}+E$, $-C_{3}-L_{3}+R_{3}+\theta W$
			Negative emergency support	$-C_{1}-L_{1}-P_{1}$, $-C_{2}-L_{2}-P_{2}$, $-C_{3}-L_{3}+R_{3}$

**Table 3:** The payment matrix under the central government’s weak emergency dispatching strategy.

				The Central Government
				Weak Emergency Dispatching
The local government	Positive emergency cooperation	The neighboring government	Positive emergency support	$-\left(1-\beta \right)C_{1}-\left(1-\eta \right)\left(1-\alpha \right)H_{1}-\left(1-\alpha \right)L_{1}+R_{1}-\left(1-\mu \right)M_{1}$, $-\left(1-\beta \right)C_{2}-\left(1-\eta \right)\left(1-\alpha \right)H_{2}-\left(1-\alpha \right)L_{2}+R_{2}-\left(1-\mu \right)M_{2}+\varpi E$, $-\varpi C_{3}-L_{3}$
			Negative emergency support	$-C_{1}-\left(1-\eta \right)H_{1}-L_{1}+R_{1}-\left(1-\mu \right)M_{1}$, $-C_{2}-L_{2}-\varpi P_{2}$, $-\varpi C_{3}-L_{3}$
	Negative emergency cooperation		Positive emergency support	$-C_{1}-L_{1}-\varpi P_{1}$, $-C_{2}-\left(1-\eta \right)H_{2}-L_{2}+R_{2}-\left(1-\mu \right)M_{2}+\varpi E$, $-\varpi C_{3}-L_{3}$
			Negative emergency support	$-C_{1}-L_{1}-\varpi P_{1}$, $-C_{2}-L_{2}-\varpi P_{2}$, $-\varpi C_{3}-L_{3}$

## Data Availability

Data sharing not applicable.
